# Effects of temperature and humidity on peritonsillar abscess volume of emergency patients

**DOI:** 10.1097/MD.0000000000031881

**Published:** 2022-12-02

**Authors:** Wei-Hsiang Su, Shiou-Shyan Yu, Tai-Ching Wu, Shih-Lun Chang

**Affiliations:** a Department of Otorhinolaryngology, Chi-Mei Medical Center, Yongkang District, Tainan, Taiwan; b Department of Radiology, Chi-Mei Medical Center, Tainan, Taiwan.

**Keywords:** hospitalization, peritonsillar abscesses, temperature

## Abstract

Climate and temperature have long been considered in relation to human diseases and mortality. In this study, we investigated whether daily temperature and humidity and patients’ personal history affect the volume of peritonsillar abscesses (PTAs). We included 52 patients with PTAs who were admitted to the emergency department of the study hospital; their computed tomography data were analyzed, and PTA volume was measured. We investigated the possible correlation between PTA volume and mean/minimum/maximum temperature and humidity. Furthermore, we obtained personal history data, including information on drinking status, smoking status, dental problems, and patients’ treatment experiences at local clinics before visiting the emergency department. The mean PTA volume was 3.93 mL, which was significantly correlated with temperature differences between 1 and 2 days before hospitalization and the day of hospitalization (*P* < .05) and also with a lack of treatment experience at local clinics (*P* < .001). However, no significant correlation was noted between PTA volume and the mean/minimum/maximum temperature and humidity on the day of hospitalization (*P* > .05). Similar findings were obtained for drinking status, smoking status, and dental problems (*P* > .1). PTA volume appears to be strongly associated with temperature differences between 1 and 2 days before hospitalization and the day of hospitalization. Patients with treatment experience at local clinics exhibited substantial increases in PTA volume. Thus, an increased PTA volume may be observed in patients who visit the emergency department without any treatment experience at local clinics or from environments that differ considerably from their current environment in terms of temperature.

## 1. Introduction

Peritonsillar abscesses (PTAs) are the accumulation of pus within the space between the tonsil and the superior constrictor muscle, near the tonsil’s superior pole. PTAs are a common deep neck infection with an incidence rate of 30 per 100000 person-years and with approximately 45000 cases occurring per year in the USA.^[1]^ The estimated yearly cost associated with PTA treatment is more than $150 million USD.^[2]^ Patients with PTAs often require emergency department admission for the treatment of severe sore throat and dysphagia. Delayed or inadequate treatment may be life threatening because the abscess may extend into the parapharynx or mediastinum.^[3]^

PTAs often occur during seasonal transitions when temperature differences increase. The close association between climate, environment, and infectious diseases in the developing world is well recognized.^[4]^ However, few studies have focused on the possible correlation between temperature and PTA volume. Therefore, in the present study, we sought to investigate this possible correlation and recommend guidelines for determining and predicting the severity of PTAs.

This observational study aimed to define the association between the temperature 1 and 2 days before hospitalization and on the day of hospitalization and the CT-based peritonsillar abscess volume of emergency patients.

## 2. Patients and methods

### 2.1. Study design and setting

This retrospective case series study was conducted at Chi Mei Medical Center, which is a tertiary referral hospital in Tainan, Taiwan. It serves approximately 2 million people, and approximately 11,000 patients are admitted to the emergency department of this hospital every month. Ethical approval for this study was obtained from the Ethics Committee of Chi Mei Medical Center (approval number: IRB0990903).

### 2.2. Population

The cases included in the present study were selected from the medical records of patients diagnosed with PTAs between July 2005 and June 2009 at Chi Mei Medical Center. PTA (code: 475) was defined on the basis of the diagnostic codes of the *International Statistical Classification of Diseases*, *Ninth Revision*.

Initially, we identified a total of 162 patients admitted to the emergency department of the study hospital. Patients with retropharyngeal abscesses, superficial craniocervical abscesses, and other tumor involvement were excluded from this study. Patients with a surgical history related to the oropharynx and those without computed tomography (CT) image data and a definitive diagnosis by an otolaryngologist were also excluded. The medical records of a total of 62 patients with PTAs were thoroughly reviewed. Data regarding the following were collected: age, sex, CT imaging, laboratory examinations, history of treatment received at local clinics, findings obtained by otolaryngologists at local clinics, patients’ personal history (drinking and smoking status, dental problems, and body mass index), and daily weather conditions. Subsequently, a total of 10 patients whose CT data indicated peritonsillar cellulites without abscess formation were excluded. Finally, the data of a total of 52 patients were analyzed in this study.

Based on the power analyses the study population collect 52 patients admitted to the emergency department. This sample size should get a power of around 82% in testing hypotheses of the primary outcome. The alpha level is 0.05 and the correlation coefficient is the measurement of effect size.

### 2.3. Temperature

The Central Weather Bureau in Taiwan provided 5-year weather data corresponding to the period for which our patient data were obtained; specifically, the data provided by Yongkang Weather Station, which is the nearest weather station to our hospital (latitude, 23°02′22″N; longitude, 120°13′43″E), were reviewed. Mean/maximum/minimum daily temperature, temperature difference, and mean daily humidity data were retrieved. We recorded the temperature 1 to 2 days before hospitalization and on the day of hospitalization.

### 2.4. Volume

Neck CT scans were performed using a 4-row spiral CT scanner (LightSpeed, GE Medical Systems). All patients underwent a neck CT (2.5-mm interval in the axial plane) performed from the skull base to the lower margin of the subclavian vein and the axial plane parallel to the infraorbitomeatal line. The digital images were transferred to the PACKS system, and the PiView system was used to analyze the images.

**Figure 1.**
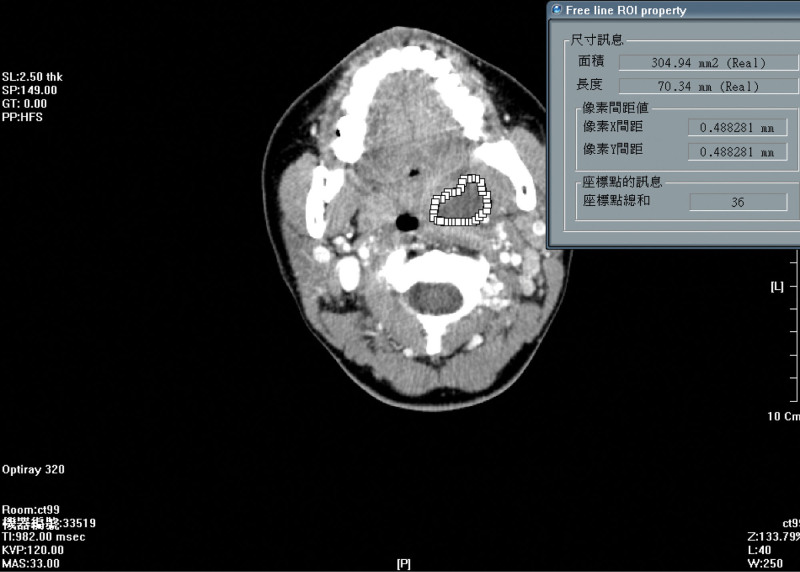
Manually drawing the outline of the left side of the peritonsillar abscess on the monitor, using the PiView STAR system, leads to a free line ROI property chart, in which the surface area measurement (304.94 mm^2^) of this image slice is available.

The summation of area technique was used to measure the total volume of the abscess. Manual drawing of the abscess outline in each image slice on the monitor helps generate a free-line region of interest property chart in the PiView system, from which the surface area can be calculated. Abscess volume can be calculated through the summation of the surface area in each image slice multiplied by the slice interval. In the present study, 2 authors, Dr Su and Yu, determined most tumor volumes and were assisted by another author, Dr Chang. A radiologist, Dr Wu, who specializes in head and neck images, was consulted when the aforementioned 2 authors differed (Fig. 1).

### 2.5. Outcome

The primary outcome is CT-based peritonsillar abscess volume of emergency patient.

### 2.6. Statistical analysis

SPSS (version 12.0, SPSS, Inc., Chicago, IL) was used to analyze descriptive statistics and perform Mann–Whitney *U* tests and Pearson product–moment correlation analyses. Differences in hospitalization day, weather, humidity, patient data, and PTA volume were juxtaposed and compared. Statistical significance was set at *P* < .05.

## 3. Results

### 3.1. Demographics

A total of 52 patients (men, 41 [79%]) were admitted to the hospital between July 1, 2005, and June 30, 2009. Most patients (95%) were aged between 17 and 71 years, and the mean age was 38.4 years (Table 1).

**Table 1:** Summary of the patients’ results (N = 52).

Variables	Mean	SD	Range
Age (yr)	38.4	16.0	8.6–87
Hospital days	4.5	1.6	1–8
Temperature grade (ºC)			
2 d before	8.24	1.98	4.4–12.3
1 day before	8.44	2.12	2.7–12.9
Hospitalization day	8.73	2.24	3.8–13.9
Average	8.47	1.73	5.8–12.2
Humidity (%)			
2 d before	69.13	5.96	53.00–88.00
1 d before	69.12	6.27	54.00–87.00
Hospitalization day	70.35	6.27	57.00–84.00
Average	69.53	5.14	56.30–85.30
Pus volume (mL)	3.93	4.51	0.25–24.88
Pus longitudinal diameter	9.35	4.15	3.00–23.00
Pus area	2.46	1.86	37.54–974.65

### 3.2. Temperature and volume

The mean PTA volume was 3.93 (0.25–24.88) mL, mean longitudinal diameter was 9.35 cm, and largest area was 2.46 cm^2^. The mean related temperature difference was 8.47°C, and the mean humidity was 69.53% (Table 1). Significant correlations were observed among temperature difference, volume, longitudinal diameter, and largest abscess area (*P* < .01; Table 2). No significant correlation was found between PTA volume and mean/maximum/minimum daily temperature or between PTA volume and mean daily humidity (Table 3).

**Table 2:** Relationships between the temperature grade 2 d before, 1 d before, and the day of hospitalization, along with the pus measures.

Variables	Temperature grade			
	2 d before	1 d before	Hospital day	Average
Pus volume	.300*	.432**	.209	.378**
Pus diameter	.221	.389**	.132	.296*
Pus area	.195	.367**	.144	.282*

Pearson product correlation

* *P* < .05;

** *P* < .01.

**Table 3:** Relationships between the humidity 2 d before, 1 d before, and the day of hospitalization, along with the pus measures.

Variables	Humidity			
	2 d before	1 d before	Hospital day	Average
Pus volume	−.204	−.100	−.170	−.188
Pus diameter	−.266	−.065	−.139	−.185
Pus area	−.190	−.057	−.189	−.173

*P < .05; **P < .01. Pearson product correlation.

### 3.3. Personal history and volume

Further analyses were performed to investigate the correlations between PTA volume and dental problems (e.g., dental caries, artificial teeth, and lacking teeth), smoking status, drinking status, body mass index, and treatment experience at local clinics. No significant correlations were found, except for treatment experience at local clinics. Before hospitalization, the mean PTA volume of patients lacking treatment experience at local clinics was 35.26 (Table 4). This value was significantly higher than that of those with treatment experience at local clinics (*P* < .001). No significant association was noted between laboratory data and PTA volume.

**Table 4:** Comparison of the patients’ risk factors in peritonsillar abscess volume (N = 52).

Variables	Number	%	Pus volume	*U*	*P*
			Mean rank		
Steroid use					
Yes	17	32.7	26.65	295.0	.961
No	35	67.3	26.43		
Teeth problems					
Yes	37	71.2	24.62	208.0	.160
No	15	28.8	31.13		
Smoking					
Yes	29	55.8	27.14	315.0	.733
No	23	44.2	25.70		
Drinking					
Yes	40	76.9	27.48	201.0	.397
No	12	23.1	23.25		
Local clinics experience					
Yes	25	48.1	17.04	101.0	.000
No	27	51.9	35.26		

Mann–Whitney *U* test.

## 4. Discussion

PTAs are a common head and neck infection. PTA incidence increases during seasonal transitions when the temperature varies substantially over a few days. Tonsillitis or pharyngitis is a preceding cause of PTA.^[3]^ Kronenberg et al^[5]^ indicated that patients with a history of recurrent tonsillitis exhibit a higher risk of recurrent PTA than do patients without any history of recurrent tonsillitis. The incidence rate of tonsillitis is the highest from January through April.^[6]^ Segal et al suggested that the predominant seasons for PTA occurrence are summer and fall, whereas Schweinfurth et al reported PTA occurrence is predominantly in spring and summer.^[7,8]^

Climate and temperature influence human diseases and mortality.^[9,10]^ However, in the present study, no significant association was noted between temperature and PTA volume; similar results were obtained for humidity. Nevertheless, temperature differences between 1 and 2 days before hospitalization and the day of hospitalization were positively correlated with the volume, square measure, and longitudinal diameter of the abscess. Temperature differences between 1 and 2 days before hospitalization and the day of hospitalization exhibited directly proportional correlations with PTA volume and longitudinal diameter.

The reasons why temperature differences affect PTA volume remain unknown. A temperature difference leads to physiological changes in the body, as was previously observed in an animal model where metabolic changes occurred in an iatrogenic abscess at different temperatures.^[11]^ Under normal circumstances, humans adapt to a cold environment by increasing peripheral vasoconstriction to maintain core body temperature. Previous findings obtained in small mammals suggest that acute cold stress suppresses several cellular and humoral components of the immune system.^[12]^ However, the findings concerning the effects of cold exposure on human immune function are inconsistent.^[13]^ The exposure of macrophages (in vitro) to a temperature of 24°C for 1 hour led to a decrease in phagocytic activity; moreover, the exposure of peritoneal macrophages (in vitro) to temperatures of 4, 10, 24, and 37°C for 1 hour suggested a close inverse association between incubation temperature and the number of cells capable of phagocytosis.^[14]^ Polderman^[15]^ stated that a decline in core body temperature causes leukocytopenia, suppressed phagocytosis, and reduced release of cytokines—factors that increase host susceptibility to infection.

We suggest that an increase in temperature difference leads to a decrease in immune function and blood supply in the peritonsillar area, thus rapidly aggravating PTAs. This phenomenon may increase PTA volume, leading to pain and other uncomfortable symptoms. Patients tend to visit hospital on the following day after the plunge of temperature. This may explain the results of our study: temperature differences between 1 and 2 days before hospitalization and the day of hospitalization exhibited a significant correlation with PTA volume (2 days before hospitalization, *P* < .05; 1 day before hospitalization, *P* < .01) Changes were noted also in the longitudinal diameter of abscesses, which may increase the likelihood of an abscess descending into the parapharyngeal space and even to the mediastinum.

Needle aspiration is a popular management option for PTA because it is a less invasive, a less painful, and the most cost-effective initial treatment approach.^[16–18]^ If ≥ 3 mL of pus is initially aspirated from a PTA, the patients should be examined again on the following day to assess the need for further aspiration.^[19]^ Repeated aspiration may be necessary in 4% to 10% of patients with PTAs.^[17,20–22]^ Because of the close association between temperature difference and PTA volume noted in our study, clinicians must consider a second needle aspiration to control the increasing PTA volume when patients have been transferred from environments that substantially differ from their current environment in terms of temperature. In cases of increased PTA volume, deep neck infections, and even mediastinitis may occur. Thus, caution must be exercised if the pus volume increases substantially.

Kilty reported that tobacco smoking is more frequent in patients with PTAs than in those with peritonsillar cellulites.^[23]^ Smoking appears to be a risk factor for PTAs.^[24,25]^ However, our study revealed no strong correlation between PTA volume and patients’ smoking status, drinking status, or dental problems. Only their treatment experience at local clinics was strongly correlated with PTA volume. Treatment received at local clinics might have facilitated treatment at our hospital for reducing or regulating PTA volume.

Although laboratory evaluation may not be necessary for PTA diagnosis, it may help gauge PTA severity and provide appropriate treatment.^[26]^ In our study, no significant correlation was noted between laboratory data and PTA volume.

Our study has some limitations. First, the study cohort did not include children because of the lack of CT image data; children are generally not exposed to radiation. Second, because of the high cost of CT, it could not be performed for all patients with PTAs, which might have introduced a bias to our findings. Finally, patients with mild PTAs were treated at their local clinics and were not referred to our hospital.

## 5. Conclusions

Global warming and weather phenomena such as El Niño affect all aspects of our lives. With the changes in climate patterns, the manifestation of PTAs may change. We found significant correlations of PTA volume with temperature differences between 1 and 2 days before hospitalization and the day of hospitalization as well as with patients’ treatment experience at local clinics. Thus, clinicians must be aware of a possible increase in PTA volume in patients lacking treatment experience at local clinics or those who are transferred from environments that differ substantially from their current environment in terms of temperature. More aggressive treatment modalities should be considered when treating these patients.

## Author contributions

**Writing—original draft:** Wei-Hsiang Su.

**Writing—review and editing:** Shiou-Shyan Yu, Shih-Lun Chang.

**Resources:** Tai-Ching Wu.
